# Characterization of Pax2 Expression in the Goldfish Optic Nerve Head during Retina Regeneration

**DOI:** 10.1371/journal.pone.0032348

**Published:** 2012-02-27

**Authors:** Marta Parrilla, Concepción Lillo, M. Javier Herrero-Turrión, Rosario Arévalo, José Aijón, Juan M. Lara, Almudena Velasco

**Affiliations:** Institute of Neuroscience of Castilla y Leon, University of Salamanca, Salamanca, Spain; Virginia Commonwealth University Medical Center, United States of America

## Abstract

The Pax2 transcription factor plays a crucial role in axon-guidance and astrocyte differentiation in the optic nerve head (ONH) during vertebrate visual system development. However, little is known about its function during regeneration. The fish visual system is in continuous growth and can regenerate. Müller cells and astrocytes of the retina and ONH play an important role in these processes. We demonstrate that *pax2a* in goldfish is highly conserved and at least two *pax2a* transcripts are expressed in the optic nerve. Moreover, we show two different astrocyte populations in goldfish: Pax2^+^ astrocytes located in the ONH and S100^+^ astrocytes distributed throughout the retina and the ONH. After peripheral growth zone (PGZ) cryolesion, both Pax2^+^ and S100^+^ astrocytes have different responses. At 7 days after injury the number of Pax2^+^ cells is reduced and coincides with the absence of young axons. In contrast, there is an increase of S100^+^ astrocytes in the retina surrounding the ONH and S100^+^ processes in the ONH. At 15 days post injury, the PGZ starts to regenerate and the number of S100^+^ astrocytes increases in this region. Moreover, the regenerating axons reach the ONH and the *pax2a* gene expression levels and the number of Pax2^+^ cells increase. At the same time, S100^+^/GFAP^+^/GS^+^ astrocytes located in the posterior ONH react strongly. In the course of the regeneration, Müller cell vitreal processes surrounding the ONH are primarily disorganized and later increase in number. During the whole regenerative process we detect a source of Pax2^+^/PCNA^+^ astrocytes surrounding the posterior ONH. We demonstrate that *pax2a* expression and the Pax2^+^ astrocyte population in the ONH are modified during the PGZ regeneration, suggesting that they could play an important role in this process.

## Introduction

The fish visual system is in continuous growth and can regenerate after suffering a lesion [1], [2]. In the peripheral growth zone (PGZ) of the retina new retinal ganglion cells (RGC) are added to the mature retina. These RGC extend their axons, which travel throughout the most vitreal part of the retina, reach the optic nerve head (ONH) and incorporate into the optic nerve in an organized way [3]. In this organized trajectory, Müller cells and astrocytes in the retina and ONH play fundamental roles. During both development and, in fish, also during continuous growth, Müller cells establish contacts with the growing axons and release different adhesion and guidance molecules, such as laminins, ephrins or immunoglobulins to the basal lamina participating in the guidance of axons through the retina [4], [5], [6]. Moreover, astrocytes in the nerve fiber layer (NFL) pack the RGC axons and seem to participate in guiding young RGC axons coming from the PGZ [7], [8]. In the ONH, both types of glial cells are involved in the formation of a favorable environment, expressing guidance molecules such as R-Cadherin, Netrin1 or Slit2, which promote the axon growth and their incorporation into the optic nerve [4], [5], [6].

After injury, in contrast to mammals, fish astrocytes promote axon regeneration and actively participate in the regenerative process [1], [9]. Müller cells are capable of dedifferentiating and originating all kinds of retinal cells recuperating the damaged region [10], [11], [12]. Moreover, they seem to produce molecules that protect the lesioned RGC during the first stages after optic nerve crush in mammals [13], [14], [15]. Astrocytes in the ONH also participate in the guidance and organization of the axons after an injury, modifying the expression of axon-guidance molecules, such as Netrin1, and packing the newly formed axons [5], [16], [17].

In vertebrates there is a population of Pax2^+^ astrocytes in the ONH during development and in adulthood [18], [19], [20]. Pax2 belongs to the transcription factor subfamily Pax2/5/8 characterized by the paired domain (PD domain) and the partial sequence homeobox as DNA binding-domains, an octapeptide that interacts with other proteins, and a transactivating/inhibiting domain [21], [22], [23]. During development it participates in the choroid fissure closure, glial precursor differentiation and RGC axon guidance throughout the ONH and the optic chiasm [24]. Moreover, Pax2 acquires a special importance in the region that originates the ONH [25], [26], [27]. In the absence of the *pax2* gene expression the choroid fissure fails to close and determines the human disease called coloboma [28]. Pax2 seems to promote the astroglial phenotype in adults, as well as participating in axon guidance processes in fish [18], [19], [20]. In some teleosts such as zebrafish (*Danio rerio*), the *pax2* gene is duplicated in two genes: *pax2a* and *pax2b*. The *pax2a* is more similar to the mammalian *pax2* gene in its expression pattern. The *pax2b* gene also participates in optic nerve development, though its expression is delayed [22].

In this work we propose to analyze the astrocyte response in the ONH after removing the PGZ, paying special attention to the Pax2^+^ astrocytes, whose function has not yet been analyzed during regeneration. Using the goldfish (*Carassius auratus*) as an animal model of central nervous system regeneration, we find that the Pax2^+^ astrocyte population and *pax2a* gene expression are affected in the absence of young RGC axons after PGZ elimination and react strongly when the regenerating axons reach the ONH again. We also compare Pax2^+^ astrocytes [19] with the S100^+^ astrocyte population in the retina and ONH, previously described [7], [8], [17], and we find that they belong to different astrocyte subpopulations. Moreover, we discover that S100^+^ astrocytes are not only involved in ONH reorganization after an injury, but also in PGZ regeneration. We also describe morphological modifications in vitreal Müller cell processes in the ONH during the whole regenerative process, suggesting that they are also involved in the guidance and reorganization of the regenerating axons. Finally, we characterize a new source of astroblasts activated after injury in the posterior ONH.

## Results

### Pax2^+^ cells in control ONH

**Figure 1:**
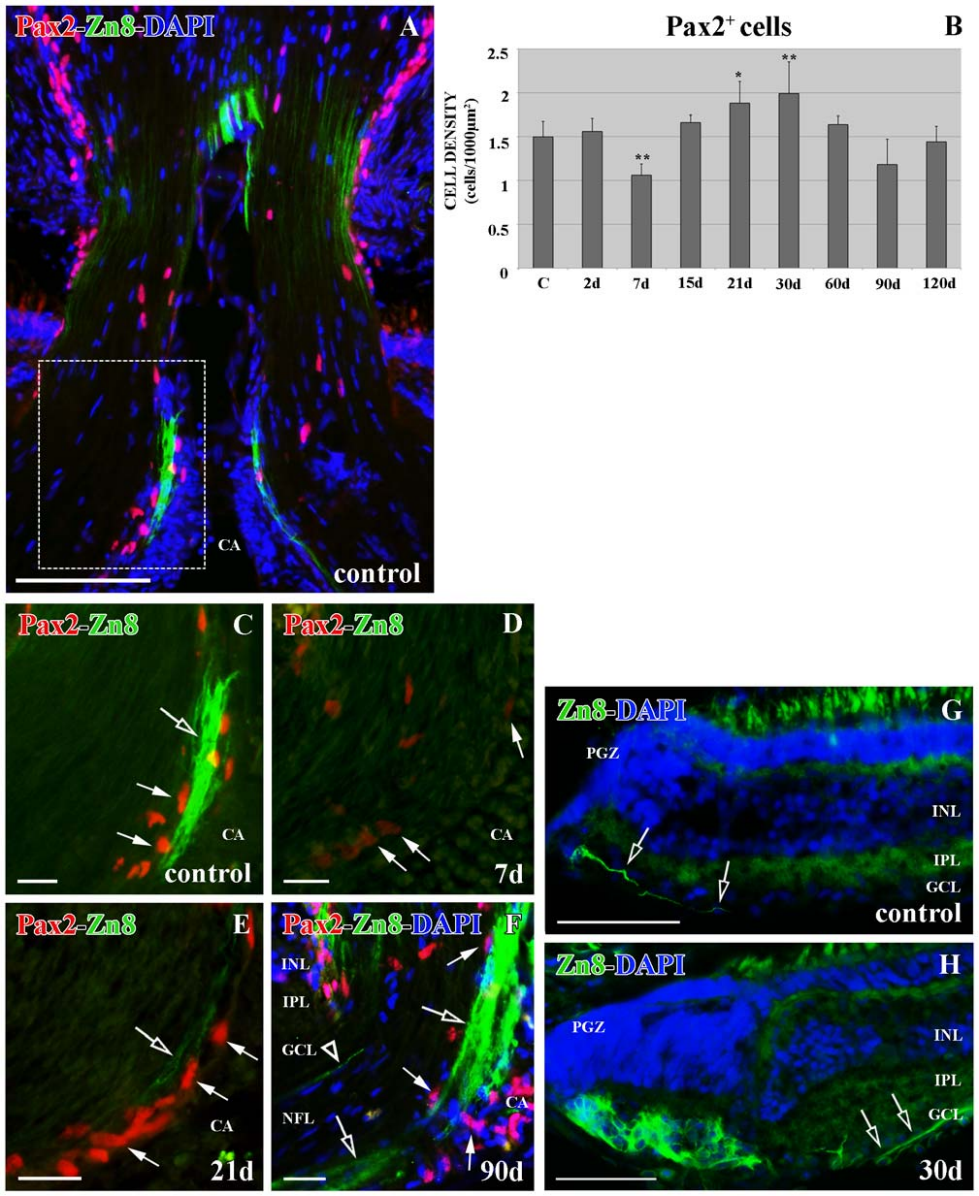
Double immunolabeling Pax2 (red)-Zn8 (green) with DAPI (blue) in the ONH. (**A**) Pax2^+^ cells are arranged close to the Zn8^+^ young RGC axons throughout the control ONH. Square enlarged in C. (**B**) Statistical analysis of the Pax2^+^ cell density in the ONH during regeneration. The asterisk indicates the significant differences (*0.05<p<0.01) and the two asterisks highly significant differences (**p<0.01). N = 4 animals per group. (**C**) Detail of the optic disc showing Pax2^+^ (arrows) close to the Zn8^+^ young RGC axons (empty arrow). (**D**) Detail of the optic disc 7 d after injury showing the Pax2^+^ cells barely labeled (arrows). (**E**) Detail of the optic disc 21 d after injury showing the Pax2^+^ cells (arrows) close to the Zn8^+^ regenerating axons (empty arrow). (**F**) Detail of the optic disc 90 d after injury showing Pax2^+^ cells (arrows) close to the Zn8^+^ regenerating axons in the growing edge (empty arrows) and in the mature ONH (empty arrowhead). (**G**) Zn8^+^ RGC axons (empty arrows) in the control PGZ. (**H**) Zn8^+^ RGC axons (empty arrows) in the PGZ 30 d post-lesion. Scale bars: A: 100 µm; C–F: 20 µm; G–H: 50 µm. CA: central artery; GCL: ganglion cell layer; INL: inner nuclear layer; IPL: inner plexiform layer; NFL: nerve fiber layer; PGZ: proliferative growth zone.

Pax2^+^ cells are exclusively located in the goldfish ONH limiting the retina and the central artery (CA) with the optic nerve (Fig. 1A). We had already characterized these Pax2^+^ cells as astrocytes [19]. They express the two astrocyte intermediate filaments: GFAP and cytokeratin, and the zonula occludens (ZO1) proteins. Moreover, they have the typical astrocyte ultrastructure features, such as a euchromatic nucleus, intermediate filaments in their cytoplasm and desmosome cell junctions [19]. Furthermore, Pax2^+^ astrocytes show a close relationship with the young RGC axons labeled with Zn8 antibody (Fig. 1A and C), which recognizes the surface molecule neurolin present in the growing axons [5].

### Pax2^+^ cells in the ONH of cryolesioned animals

In this work we analyzed the Pax2^+^ astrocyte response in the goldfish ONH after PGZ cryolesion. Although Pax2^+^ cell distribution in the ONH was similar to that of controls during the whole regenerative process (Figs. 1A–F), we found modifications in number and immunolabeling features.

At 2 d post-cryolesion, there was no difference in the Pax2^+^ astrocyte number compared with control animals (p>0.05) (Fig. 1B). Nevertheless, at 7 d post-cryolesion, Zn8^+^ growing RGC axons were not detected (Fig. 1D), and coincident with this, Pax2 immunolabeling was much weaker than in control animals (Figs. 1C–D) and there was a highly significant decrease in the number of Pax2^+^ cells (**p<0.01) (Fig. 1B). The intensity of labeling in Pax2^+^ cells was recovered once the first Zn8^+^ regenerating axons reached the ONH at 15 (data not shown)-21 d after cryolesion (Fig. 1E). Furthermore, at this time after injury there was an increase in the number of Pax2^+^ cells, which was highly significant (**p<0.01) at 30 d (Fig. 1B). From 90 d after injury we found a large amount of regenerating RGC axons arriving at the ONH (Fig. 1F). This coincided with the high quantity of Zn8^+^ growing RGC axons immunodetected in the PGZ (Fig. 1G). Pax2^+^ cell labeling was similar to that of control animals (Figs. 1C, F), as well as their number from 60 d until the 120 d post-injury stages analyzed in this work (Fig. 1B).

We combined double immunolabeling of Pax2 with the astrocyte antibodies anti-intermediate filaments, GFAP and cytokeratin, and the ZO1 protein as we did previously in control animals [19]. We did not find differences in Pax2^+^ cell characterization, nor in GFAP, cytokeratin or ZO1 protein distribution compared with controls (not shown).

**Figure 2:**
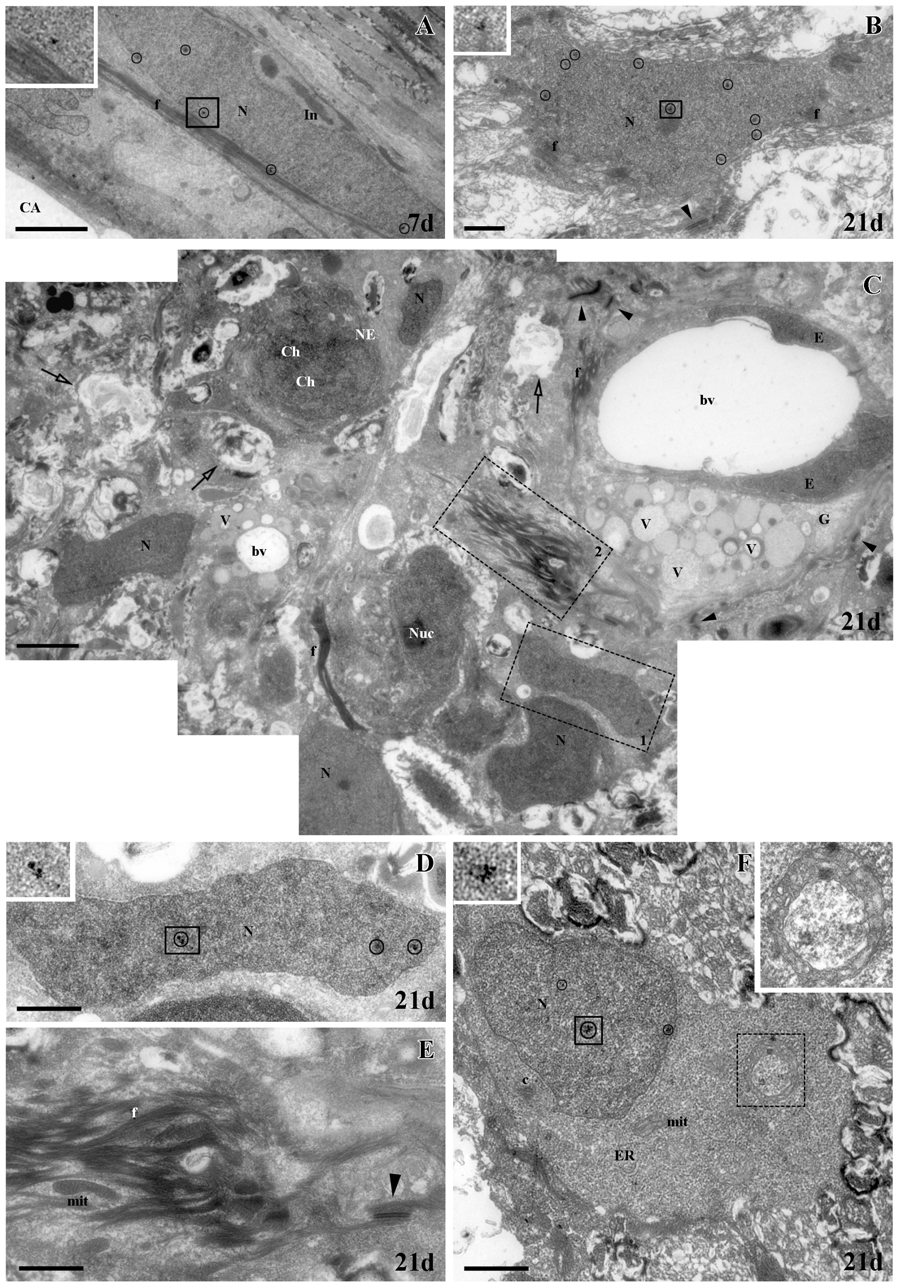
Immunogold electron microscopy labeling of Pax2 in the ONH. (all gold particles have been circled). (**A**) Pax2^+^ astrocyte with euchromatic nucleus (N) at 7 d post-injury. Inset: gold particle from the square. (**B**) Pax2^+^ astrocyte with euchromatic nucleus (N) at 21 d post-injury. Inset: gold particles from the square. (**C**) Composition of micrographs showing numerous glial cells (N: nucleus) among astrocyte processes with intermediate filaments (f), areas in degeneration (empty arrows) and dividing cells (ch: chromosomes) at 21 d post-injury. Close to the blood vessel (bv) there are granulocytes (G). Squares 1 and 2 are enlarged in D and E respectively. (**D**) Detail of a Pax2^+^ cell with a euchromatic nucleus (N). Inset: gold particles from the square. (**E**) Detail of intermediate filament packages (f). (**F**) Pax2^+^ astroblast with euchromatic nucleus (N). Upper left corner inset: gold particles from the bold square. Dotted square enlarged in upper right corner showing a doughnut-shaped mitochondria. Scale bars: A, C: 2500 nm; B, D–F: 1000 nm. Arrowheads: desmosomes; c: centrioles; CA: central artery; E: endothelium; ER: rough endoplasmic reticulum; f: intermediate filaments; In: indentations; mit: mitochondria; NE: nuclear envelope; Nuc: nucleolus; V: vesicles.

To further study the modifications and changes in the cell typology of Pax2^+^ cells after cryolesion, we analyzed their ultrastructure by immuno-electron microscopy. At 7 d and 21 d after the cryolesion, we found Pax2 labeling located precisely in the nucleus of some cells with the typical ultrastructural features of astrocytes (Figs. 2A–B), similar to that of control animals [19]. They showed a euchromatic nucleus with indentations, they had packs of intermediate filaments in the cytoplasm and they joined to each other by numerous desmosomes (Figs. 2A–B). As we described previously in control animals [19], we also found Pax2^−^ astrocytes (not shown). At 21 d post-injury, in contrast to control and 7 d, we also found numerous cells with euchromatic nuclei, and some of them showed Pax2 immunolabeling (Figs. 2C–D). These are probably astrocytes. It is noteworthy that surrounding all these cells there were a great number of astrocyte processes (Figs. 2C, E), adding another characteristic to the astrocyte reaction. In accordance with this, we also found numerous regions in degeneration (Fig. 2C) and granulocyte cells, with dark and light vesicles in their cytoplasm, associated with the blood vessels (Fig. 2C). Dividing cells (Fig. 2C) and Pax2^+^ immature cells, probably astroblasts (Fig. 2F), were also found. Interestingly, in all dividing and immature cells, doughnut-shaped mitochondria were detected in their cytoplasm (Fig. 2F).

### Molecular characterization of goldfish Pax2a and phylogenetic analysis

**Figure 3:**
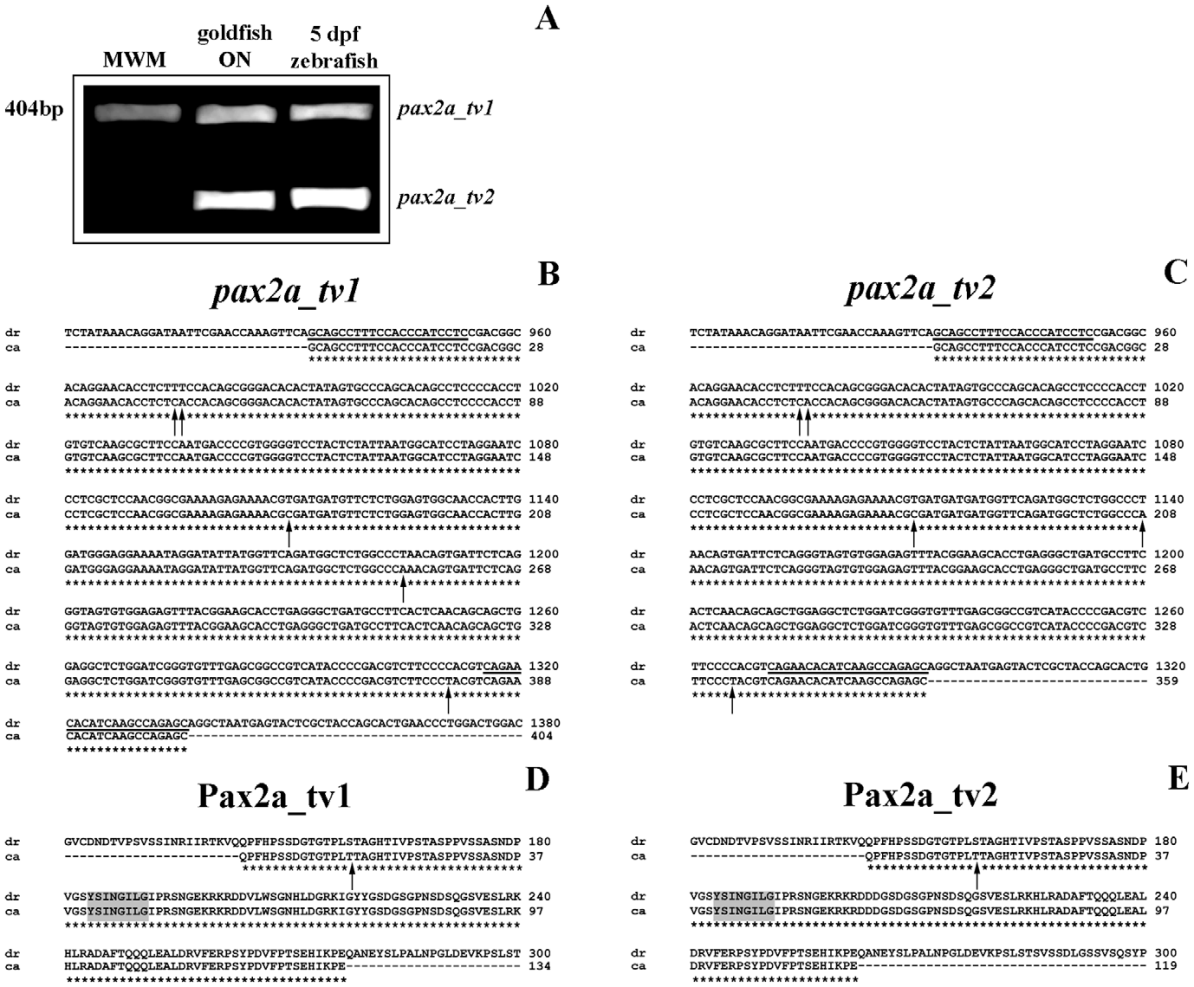
Gene expression and molecular characterization of goldfish *pax2a* mRNAs. (**A**) Expression of two *pax2a* mRNA variants (*pax2a_tv1* and *pax2a_tv2*) in goldfish optic nerve and 5 dpf zebrafish embryos by RT-PCR. MWM: molecular weight marker. (**B–C**) Comparison of two goldfish partial-length cDNAs, *pax2a_tv1* and *pax2a_tv2*, with their corresponding homologous in zebrafish. Alignment produced with the ClustalW program of the nucleotide sequences of *pax2a* of both cyprinidae species. The primers used for the molecular characterization are underlined and indicated in Table 2. The differences in the base pairs are shown with an arrow and the similarities with an asterisk. (**D–E**) Comparison of goldfish Pax2a_tv1/tv2 and their corresponding homologous in zebrafish. Alignment produced with the ClustalW program of the deduced amino acid sequences of Pax2a_tv1/tv2 of both cyprinidae species. The difference in the amino acids is shown with an arrow and the similarities with an asterisk. The octapeptide domain is marked in grey.

To analyze the goldfish *pax2a* gene expression level in ONH during regeneration using RT-qPCR assays, firstly, two partial-length cDNAs of 404 bp and 359 bp were cloned (Fig. 3A). Sequence analysis encoding both cDNAs showed two open reading frames (ORF) of 134 and 119 amino acids, respectively, which include the Pax2a octapeptide domain. Comparing these two goldfish cDNAs with other *pax2a* isoforms identified for alternative splicing in zebrafish (*pax2a_tv1*: ENSDART00000076992 and *pax2a_tv2*: NM_131184.2 [29], in Fig. 3B–C), we found that both goldfish transcripts are highly homologous to 11 isoforms of zebrafish (98% of identity) and we denominated the new isoforms characterized in this work *pax2a_tv1* and *pax2a_tv2*. What is more, as shown in Figs. 3D–E, the comparison of each Pax2a isoform of goldfish with its corresponding zebrafish homolog showed a 99% of identity with only a single change of a threonine in goldfish instead of a serine in zebrafish.

**Figure 4:**
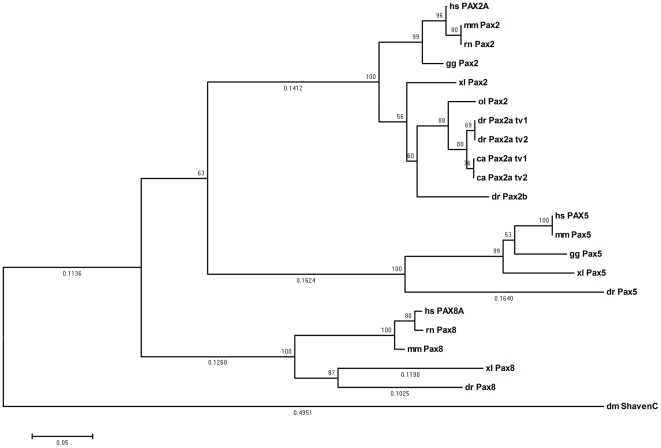
Phylogenetic analysis generated by the NJ method using MEGA4 [**66**] from the Pax2/5/8 protein family sequences. Whole numbers indicate bootstrap values of 1000 replicates and branch distances are given in decimal numbers when >50%. *Drosophila melanogaster* Shaven, Pax5 and Pax8 proteins were used as out groups. Organisms from which each protein sequence was derived are indicated in the prefix, ca, goldfish (*Carassius auratus*); dr, zebrafish (*Danio rerio*, Pax2a_t2: NP_571259.1, Pax2a_t1: ENSDART00000076992, Pax2b: NP_571715.1, Pax5: NP_571713.1, Pax8: XP_001339893.3); ol, Japanese medaka (*Oryzias latipes*,Pax2: CAB09696.1); xl, African clawed frog, (*Xenopus laevis*, Pax2: NP_001079830.1, Pax5: NP_001079237.1, Pax8: NP_001081941.1); hs, human (*Homo sapiens*, PAX2A: NP_003978.2, PAX5: NP_057953.1, PAX8A: NP_003457.1); mm, mouse (*Mus musculus* Pax2: NP_035167.3, Pax5: NP_032808.1, Pax8: NP_035170.1); rn, rat (*Rattus norvegicus*, Pax2: NP_001099831.1, Pax8: NP_112403.2); gg, chicken (*Gallus gallus*, Pax2: NP_990124.1, Pax5: NP_989755.1); and dm, fruit fly (*Drosophila melanogaster*, Shaven: NP_726645.3).

In order to analyze the phylogeny of the two goldfish *pax2a* transcript variants found in this work, using the neighbour-joining (NJ) method, a phylogenetic tree of the aligned amino acid sequences from Pax2 of different representative species was constructed (Fig. 4). We included two other members of the Pax2/5/8 subfamily (Pax5 and Pax8) [30] and the *Drosophila melanogaster* Shaven [31], [32] sequence, which were used as out groups in this analysis. We found that both isoforms of Pax2a of goldfish and zebrafish belong to the same clade and they are separated from the Pax2 of other vertebrates. In addition, the ancestral *D. melanogaster* Shaven is found in a basal position compared to the rest of the members of the Pax2/5/8 subfamily in the tree (Fig. 4). Thus, we demonstrate that transcripts of goldfish *pax2a* are orthologous to the rest of vertebrate *pax2*.

### Expression analysis of goldfish Pax2a of the ONH

**Figure 5:**
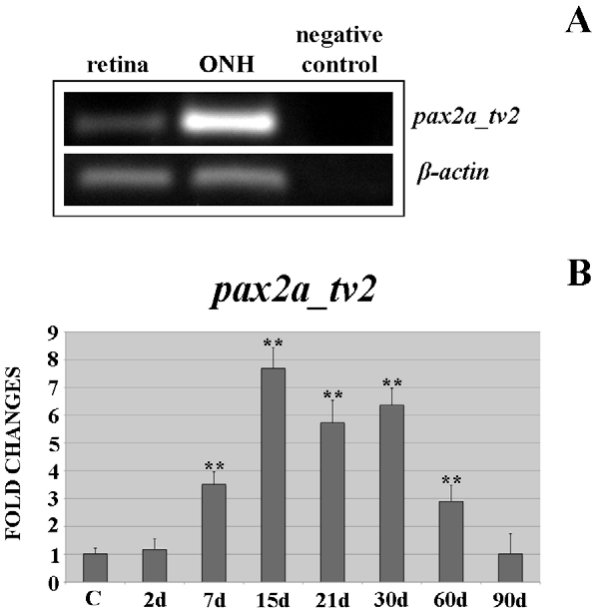
Differential gene expression of goldfish *pax2a_tv2* mRNA. (**A**) Expression of *pax2a_tv2* mRNA in goldfish retina and ONH by RT-PCR. PCR amplification of zebrafish and goldfish *β-actin* mRNAs were used as internal controls. (**B**) Fold changes of gene expression of *pax2a_tv2* in the goldfish ONH during regeneration. The highly significant differences are indicated with two asterisks (**p<0.001).

As most *pax2a* studies in zebrafish are carried out with the transcript variant *pax2a_tv2*, which is orthologous to the rest of vertebrate *pax2*, we chose goldfish *pax2a_tv2* to analyze its expression during regeneration. As shown in Fig. 5A, we previously checked goldfish *pax2a_tv2* expression in control retina and ONH using RT-PCR assays. We found that *pax2a_tv2* is expressed in both; however, it is noteworthy that gene expression level in retina is lower than in the ONH.

Moreover, in RT-qPCRs, we found changes in *pax2a_tv2* expression levels during regeneration after cryolesion (Fig. 5B). At 2 d post-injury, we did not detect differences in *pax2a_tv2* expression compared with controls (p>0.05). Nonetheless, from 7 d (3.51 fold) to 30 d after cryolesion (2.90 fold) its gene expression level was highly significantly increased (**p<0.01) compared with control animals (1 fold). The *pax2a_tv2* expression level reached the maximum at 15–30 d regenerative stages (15 d: 7.68 fold; 21 d: 5.72 fold; 30 d: 6.36 fold), anticipating the arrival of axons at the ONH at 21 d post-injury (Fig. 1E). Finally, at 90 d post-injury (1.01 fold) *pax2a_tv2* expression was similar to control levels (p>0.05).

### S100^+^ cells in control retina

**Figure 6:**
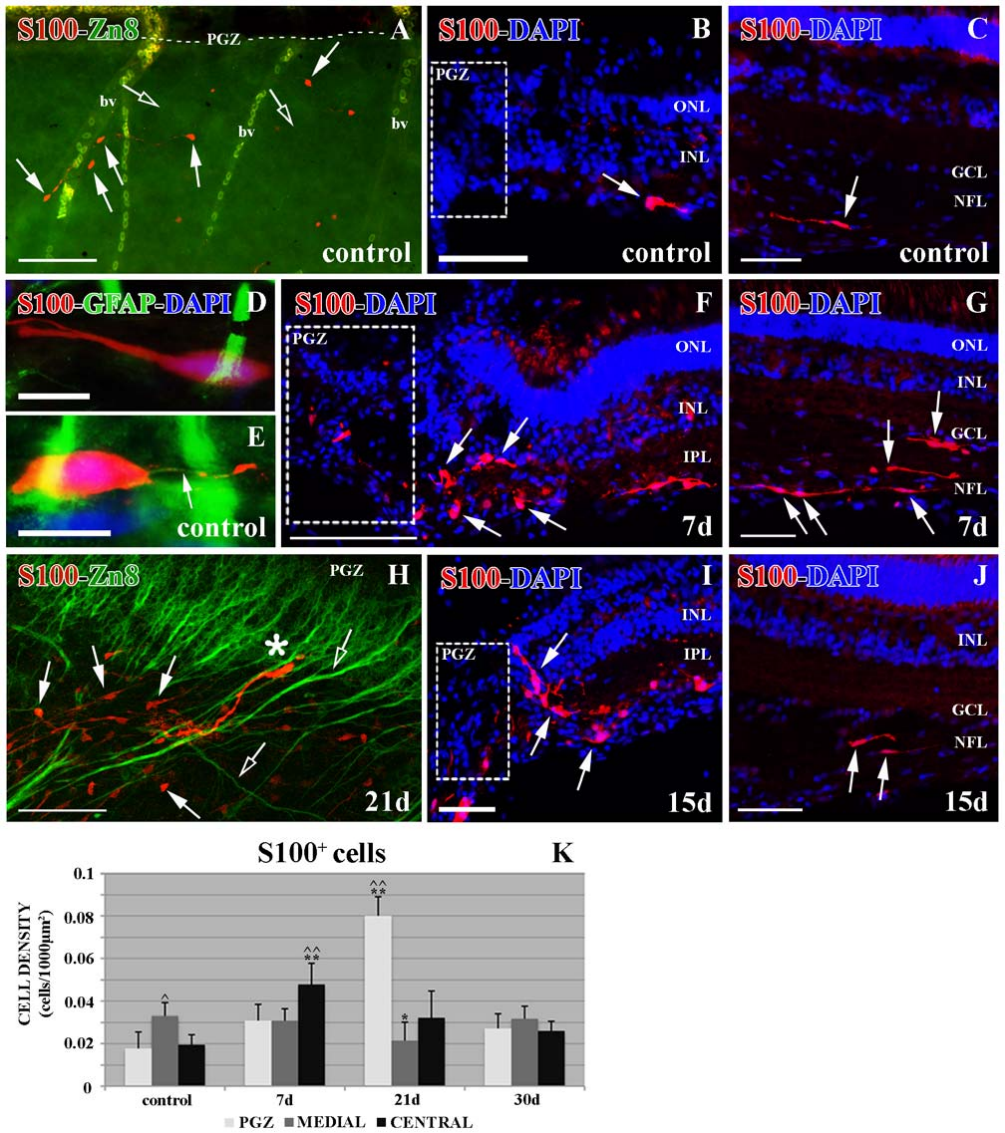
Double immunolabeling S100 (red)-Zn8 (green) and S100 (red)-GFAP (green) with DAPI (blue). (**A**) S100^+^ cells (arrows) and Zn8^+^ growing axons (empty arrows) in an *in toto* control retina, bv: blood vessels. PGZ: where this area is in the picture (broken line). (**B**) S100^+^ cell (arrow) in a control retina close to the PGZ. (**C**) S100^+^ cell (arrow) in control retina close to the ONH. (**D**) Detail of an S100^+^/GFAP^−^ cell in the control retina. (**E**) Detail of an S100^+^ cell with GFAP^+^ processes (arrow) in the control retina. (**F**) S100^+^ cells (arrows) in the injured PGZ at 7 d post-cryolesion. (**G**) High quantity of S100^+^ cells (arrows) in the retina close to the ONH at 7 d post injury. (**H**) S100^+^ cells (arrows) and Zn8^+^ regenerating axons (empty arrows) in an *in toto* retina 21 d after the cryolesion. The asterisk indicates an S100^+^ cell directing its processes to the inner retina. Confocal, maximum projection of 18 optical planes acquired with a step size of 1 micron. A 40× oil inmersion objective with a N.A. of 1.25 was used and the pinhole was set to 1.20 Airy units. (**I**) S100^+^ cells (arrows) in the injured PGZ at 15 d post-cryolesion. PGZ: where this area is in the picture. (**J**) S100^+^ cells (arrows) in the retina close to the ONH 15 d post injury. (**K**) Statistical analysis of the S100^+^ cell density in the retina during regeneration. The cap and two caps indicate the significant (∧0.01<p<0.05) or highly significant (∧∧p<0.01) differences among the three regions in control and regeneration retinas; the asterisk or two asterisks mean that the differences in each region of the retina between the injured and controls are significant (*0.01<p<0.05) or are highly significant (**p<0.01), respectively. N = 5 animals per group. Scale bars: A: 100 µm; B–C, F–G, I–J: 50 µm; D–E: 10 µm; H: 75 µm. GCL: ganglion cell layer; INL: inner nuclear layer; IPL: inner plexiform layer; NFL: nerve fiber layer; ONL: outer nuclear layer; PGZ: proliferative growth zone.

To better analyze the astrocyte response after PGZ cryolesion in the ONH, we used the typical astrocyte marker S100. We found that S100^+^ cells were distributed throughout the NFL of the retina from the PGZ (Fig. 6A–B) to the central retina close to the ONH (Fig. 6C) in control animals. S100^+^ cells did not show any close relationship with either young Zn8^+^ RGC axons or retinal blood vessels (Fig. 6A). Nevertheless, the number of S100^+^ cells was higher in the intermediate zone than in the PGZ and the central retina (∧0.01<p<0.05) (Fig. 6K).

Some retinal S100^+^ cells had weak GFAP^+^ labeling in their processes, which never co-localized with the S100 immunolabeling (Figs. 6D–E). Both S100^+^/GFAP^−^(Fig. 6D) cells and S100^+^/GFAP^+^ cells (Fig. 6E) showed the same elongated morphology and location in the NFL, suggesting that both of them are astrocytes, as previously proposed for tench [8].

### S100^+^ cells in cryolesioned retina

At 7 d after cryolesion, the PGZ was completely disorganized (Fig. 6F) and we did not find Zn8^+^ young RGC axons (data not shown). In this stage of degeneration, the S100^+^ cell population changed. In the PGZ, they were specially located close to the injured zone showing a round morphology (Fig. 6F), but there was no significant increase in their density (p>0.05) (Fig. 6K). On the other hand, in the central retina, there was a highly significant increase in their density compared with controls (**p<0.01) (Fig. 6K). These S100^+^ cells maintained their elongated morphology and were distributed throughout the whole NFL (Fig. 6G).

At 15–21 d after cryolesion, a high amount of regenerating RGC axons was labeled with the anti-Zn8 antibody in the PGZ (Fig. 6H). In this region we found a highly significant increase of S100^+^ cells (**p<0.01) (Fig. 6K). Some of these S100^+^ cells were closely located to the Zn8^+^ regenerating axons, but other S100^+^ cells were not related with them (Fig. 6H). However, some S100^+^ cells migrated or directed their processes to the inner layers of the retina (Figs. 6H–I) establishing a limit between the damaged retina and the intact retina (Figs. 6I).

The density of S100^+^cells in the central retina was not significantly different from control retinas (p>0.05) (Fig. 6K) and the cells maintained their elongated morphology (Fig. 6J). Meanwhile, the number of S100^+^ cells in the intermediate zone suffered a significant decrease (*0.01<p<0.05) compared with controls (Fig. 6K).

From 30 d post-injury, the PGZ started to regenerate (Fig. 1G) and the number of S100^+^ cells reached the control levels (p>0.05) in all regions of the retina (Fig. 6K).

### S100^+^ cells in control ONH

**Figure 7:**
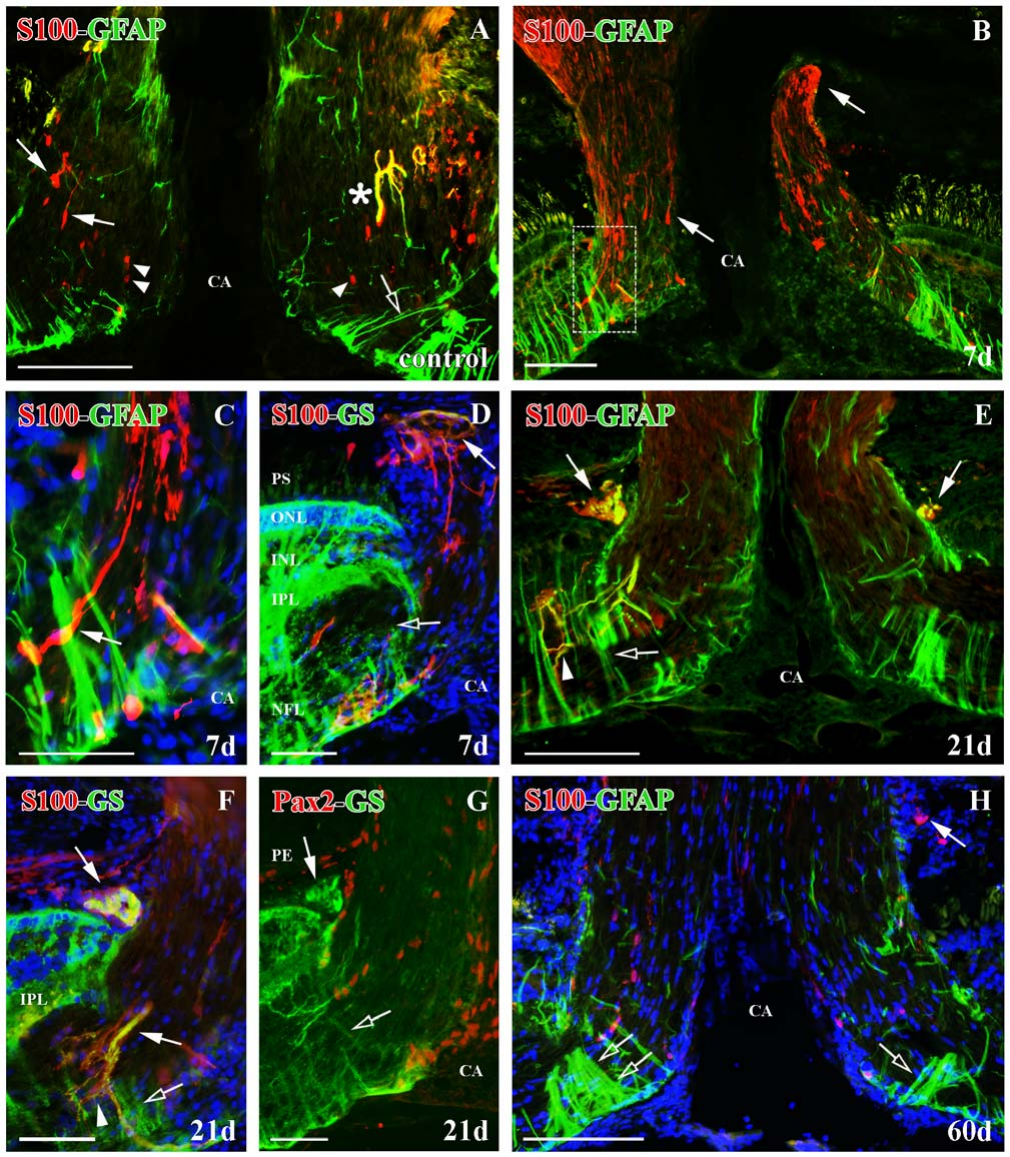
Double immunolabeling S100 (red)-GFAP (green), S100 (red)-GS (green) and Pax2 (red)-GS (green) with DAPI (blue) in the ONH. (**A**) S100^+^ astrocytes (arrows), S100^+^/GFAP^+^ astrocyte (asterisk), S100^+^ oligodendrocytes (arrowheads) and GFAP^+^ Müller cell vitreal processes (empty arrow) in control goldfish. (**B**) S100^+^ astrocytes with long processes at 7 d post-injury (arrows). Square enlarged in C. (**C**) Detail of the S100^+^ astrocytes with long processes (arrow) in the optic disc (**D**) S100^+^/GS^+^ astrocytes surrounding the posterior part of the ONH (arrow) and GFAP^+^ Müller cells with disorganized vitreal processes (empty arrow) at 7 d post-injury. (**E**) S100^+^/GFAP^+^ astrocytes surrounding the posterior part of the ONH and in the optic disc (arrows), S100^+^/GFAP^+^ processes (arrowhead) and GFAP^+^ Müller cells with disorganized vitreal processes (empty arrow) at 21 d post-injury. (**F**) S100^+^/GS^+^ astrocytes surrounding the posterior part of the ONH and in the optic disc (arrows), S100^+^/GS^+^ processes (arrowhead) and GS^+^ Müller cells with disorganized vitreal processes (empty arrow) at 21 d post-injury. (**G**) Pax2^−^/GS^+^ astrocytes surrounding the posterior part of the ONH (arrows) and GS^+^ Müller cells vitreal processes (empty arrow) at 21 d post-injury. (**H**) Few S100^+^ astrocytes are surrounding the posterior part of the ONH (arrow) and there is a great amount of GFAP^+^ Müller cell vitreal processes (empty arrows) at 60 d post-injury. Scale bars: A–B, E, H: 100 µm; C–D, F–G: 50 µm. CA: central artery; INL: inner nuclear layer; IPL: inner plexiform layer; NFL: nerve fiber layer; ONL: outer nuclear layer; PE: pigmentary epithelium; PS: photoreceptor segments.

In the ONH of control animals, S100^+^ cells were scarce and they had an elongated shape and processes similar to those previously described in retina (Fig. 7A). S100^+^ cells were mainly located longitudinal to the RGC axons and they did not limit the optic nerve with the CA and the retina (Fig. 7A). Moreover, few of them showed co-localization with the astrocyte marker GFAP (Fig. 7A). We also found a few S100^+^cells with rounded and small soma without processes, mainly located in the optic disc and always negative to GFAP. It has been suggested that they may be oligodendrocytes (Fig. 7A) [33], [34].

### S100^+^ cells in the ONH of cryolesioned animals

At 7 d after cryolesion, we found a high increase in S100^+^ processes in the whole ONH (Figs. 7B–D). S100^+^ cells located between the RGC axons maintained their elongated shape but they presented longer processes compared with controls (Fig. 7C). In the posterior part of the ONH limiting with the choroids and sclera, some S100^+^ cells reacted strongly to the injury (Figs. 7B, D). They were barely detected in control animals. These cells, whose somata are located in the glia limitans forming a ring, direct their processes transversally to the inner ONH (Fig. 7D) and they also co-express glutamine synthetase (GS) in their cytoplasm (Fig. 7D). We did not see any change in the S100^+^/GFAP^+^ cell population located in the ONH (not shown).

At 15–21 d after the cryolesion, the appearance of S100 labeling among the RGC axons was similar to that of control animals (Figs. 7E–F) and was maintained until the latest regenerative stages analyzed in this work (Fig. 7H). However, we detected strongly labeled S100^+^ processes, which leave from the inner part of the inner plexiform layer (IPL) and they are addressed to the optic nerve longitudinal to the RGC axons (Figs. 7E–F). These processes were also positive for GFAP (Fig. 7E) and GS (Fig. 7F). In the posterior ONH, the S100^+^ ring was conserved in this regenerative stage and co-localizes with GFAP (Fig. 7E) and GS (Fig. 7F). The same structure appeared Pax2^−^ (Fig. 7G). This suggests that there are two populations of astrocytes that react to the injury.

S100^+^ cells recovered an organization similar to that of control animals, including the S100^+^ ring located in the posterior ONH, from 30 d post-injury onwards (Fig. 7H).

During the whole regenerative process, we did not find changes in the S100^+^ cells with round-shape and small soma (not shown).

### Müller cells surrounding the ONH

Fish retina Müller cells have been clearly characterized as GFAP^+^/GS^+^ in control animals [7], [19]. Those located surrounding the ONH also have special morphological features and establish a limit between the retina and the optic nerve in the optic disc [7].

In the first regenerative stages (7–21 d) we did not find many GFAP^+^/GS^+^ Müller cell vitreal processes surrounding the ONH (Figs. 7D–G). In contrast, at 30–90 d post-injury, a high number of these Müller processes were detected surrounding the ONH (Fig. 7H). This was coincident with the massive arrival of regenerating RGC axons in this region, some of them located far from the growing edge (Figs. 1E, G).

### Dividing cells in the ONH in cryolesioned animals

**Figure 8:**
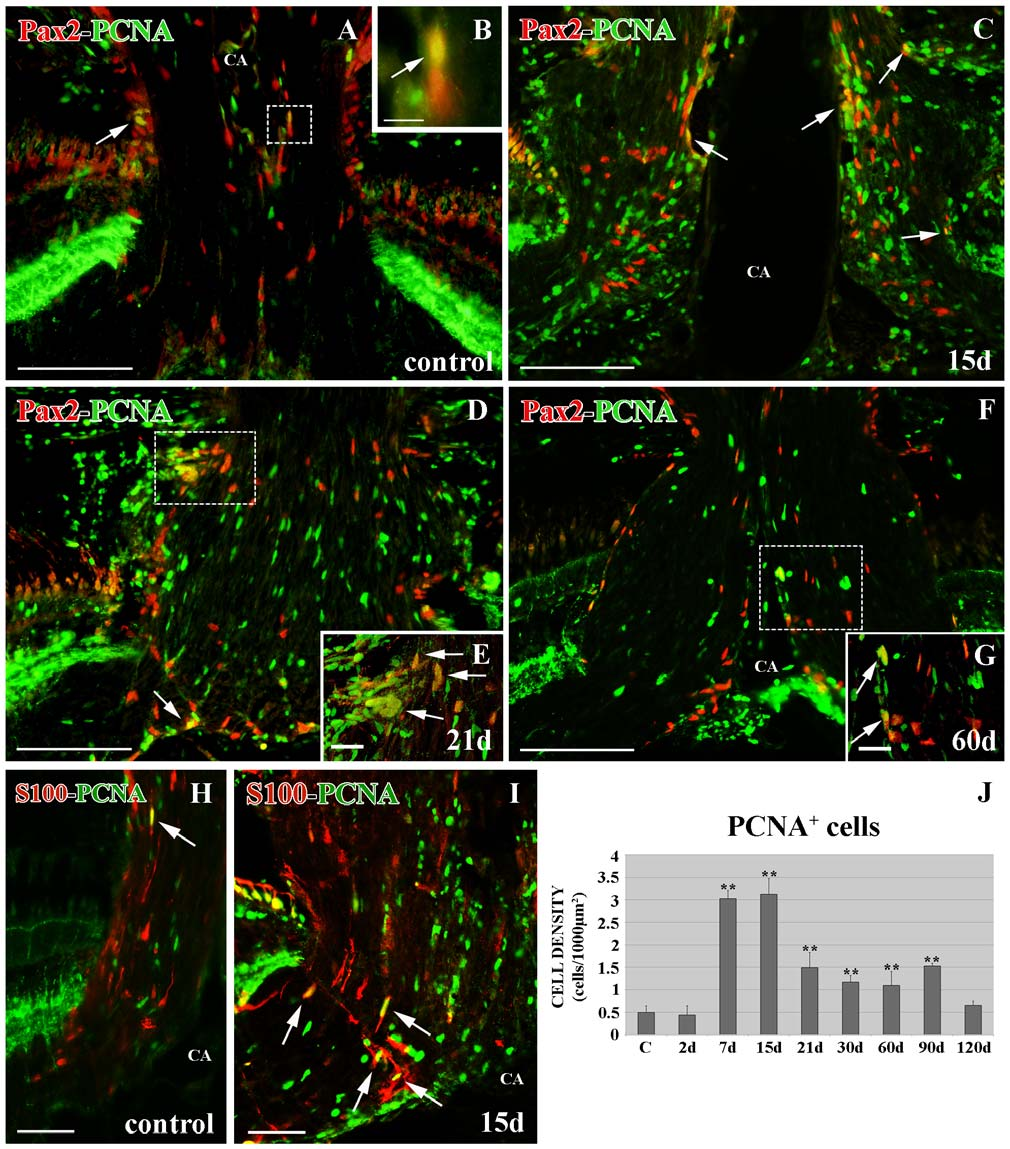
Double immunolabeling Pax2 (red)-PCNA (green) and S100 (red)-PCNA (green) in the ONH. (**A**) In control retinas there are scarce Pax2^+^/PCNA^+^ cells (arrow) close to the limit of the ONH with the retina in controls. Square enlarged in B. (**B**) Detail of a Pax2^+^/PCNA^+^ cell (arrow) close to the CA. (**C**) Pax2^+^/PCNA^+^ cells (arrows) close to the CA and the limit of the ONH with the retina after 15 d of lesion. (**D**) Pax2^+^/PCNA^+^ cells (arrows) after 21 d of lesion. Square enlarged in E. (**E**) Pax2^+^/PCNA^+^ cells (arrows) close to the limit of the ONH with the retina at 21 d after injury. (**F**) Pax2^+^/PCNA^+^ cells after 60 d of injury. Square enlarged in G. (**G**) Pax2^+^/PCNA^+^ cells (arrows) close to the CA. (**H**) Scarce S100^+^/PCNA^+^ cells (arrow) in control ONH. (**I**) S100^+^/PCNA^+^ cells (arrows) between the RGC axons at 15 d post-injury. (**J**) Statistical analysis of PCNA^+^ cell density in the ONH during regeneration. The two asterisks indicate the highly significant differences (**p<0.01). N = 4 animals per group. Scale bars: A, C, D, F: 100 µm; B, E, G: 10 µm; H–I: 50 µm. CA: central artery.

We also analyzed the number of proliferative cells labeled with PCNA during the whole regenerative process in the ONH. Similar to Pax2^+^ cells, the PCNA^+^ cell number did not undergo significant changes at 2 d after cryolesion (p>0.05) compared with controls (Fig. 8J). However, from 7 d to 90 d post-injury, the number of PCNA^+^ cells was highly significant (**p<0.01) compared with controls (Fig. 8J). The normal number of cells (p>0.05) was reached at 120 d post-cryolesion (Fig. 8J).

In order to know which of these PCNA^+^ cells were Pax2^+^ astrocytes, we performed immunolabeling. From 15 d to 60 d after cryolesion (Figs. 8C–G), we found more Pax2^+^/PCNA^+^ cells than in control animals (Figs. 8A–B). However, the majority of the PCNA^+^ cells were negative to Pax2. The Pax2^+^/PCNA^+^ cells found were part of the glia limitans of the central artery (CA) (Figs. 8C, F–G), but at earlier regenerative stages, they were mostly located in the posterior limit between the retina and the optic nerve forming a ring around the ONH (Figs. 8D–E). We did not find Pax2^+^/PCNA^+^ cells among the RGC axons.

To analyze if there was some S100^+^ cell renewal, we also carried out double immunolabeling with PCNA. We found more S100^+^/PCNA^+^ cells than in control animals especially present at 15–21 d post-injury (Figs. 8H–I). However, similar to the results described for Pax2^+^/PCNA^+^ cells, we found few S100^+^/PCNA^+^ cells among the whole PCNA^+^ cell population during regeneration (8H). These S100^+^/PCNA^+^ cells were always located between the RGC axons but not forming the glia limitans (Fig. 8I).

## Discussion

To our knowledge this is the first report analyzing the Pax2^+^ astrocyte population and the *pax2a* gene expression in the ONH during regeneration of the RGC axons. In addition, with the combination of different astrocyte markers we differentiate the Pax2^+^ astrocyte population from S100^+^ astrocytes, which have a different location in the ONH and extend throughout the retina. Moreover, they react differently during the degeneration and regeneration of young RGC axons. This suggests that Pax2^+^ astrocytes and S100^+^ astrocytes belong to different glial subpopulations in the ONH.

### Pax2^+^ and S100^+^ cells in control animals in the ONH

We demonstrate, that the Pax2 protein is exclusively located in a subpopulation of astrocytes in the ONH control [19] and during regeneration. Recent studies have demonstrated that Pax2 expression is maintained in adult vertebrate retina and ONH and they suggest that this transcription factor is involved in the astrocyte phenotype maintenance [18], [19], [20].

We find that S100^+^cells may belong to a different glial subpopulation than Pax2^+^ astrocytes. The S100^+^ cells are firstly located in the NFL and ONH in contrast to the Pax2^+^ astrocytes, which are exclusively located in the ONH [19]. Moreover, S100^+^ cells are preferably arranged longitudinal to the RGC axons probably packing them as they do in tench [7]. However, Pax2^+^ astrocytes are mostly located limiting the retina and CA with the ONH and they co-localize with GFAP [19]. Furthermore, S100^+^ astrocytes are not closely located to newly formed RGC axons in the retina and ONH, in contrast to Pax2^+^ astrocytes [19]. Also, S100^+^ astrocytes are not related with blood vessels in contrast to Pax2^+^ astrocytes in the goldfish optic nerve, which limit the blood vessels [19] and retinal Pax2^+^ astrocytes in mammals, which are highly involved in retina angiogenesis [35], [36].

### Pax2^+^ and S100^+^ cells in lesioned animals

In the ONH after injury, we find a different response of Pax2^+^ with regard to S100^+^ astrocytes after the first days post-cryolesion. In the absence of young axons, the number of Pax2^+^ cells decreases as well as the immunolabeling intensity. This maybe a strong response of the S100^+^ astrocyte population in the goldfish ONH as described in tench [17]. This decrease of Pax2^+^ cells could be due to the cell death led by the axon degeneration, as demonstrated in mammals [37]. However, we cannot discard the possibility that the Pax2 protein changes its phosphorylation level [38], [39] not being detectable by the antibody [40]. In accordance with this last supposition, we find that *pax2a* mRNA levels are 3.5 fold elevated at 7 d post-injury, showing that although the protein is not detectable, the gene is highly transcribed. The arrival of the young axons to the ONH is coincident with the increase of Pax2^+^ astrocytes reaching higher levels than in control animals. Previous studies during mouse, zebrafish and chicken development demonstrate that Pax2 is directly involved in the RGC axon guidance throughout the ONH and optic chiasm [25], [27], [41]. It is known that astrocytes play a fundamental role guiding, organizing and packing the regenerating axons [9], [17], [34]. Probably the Pax2^+^ astrocyte increase in the ONH is related with those processes.

However, the S100^+^ cells increase during the first days of lesion. With regard to the retina, the S100^+^ increase their number when newly formed RGC in the PGZ send their growing axons to the optic nerve. This high quantity of S100^+^ cells is probably due to their migration from the medial retina, where their number decreases at the same regenerative stage. Although, we do not discount the idea that some S100^+^ cells divide and increase their number, most of them did not show co-labeling with PCNA (not shown). S100 protein is involved in numerous intracellular and extracellular processes potentially active in regeneration [42], [43]. Thus, its intracellular activity in the cytoskeleton dynamic may explain the high amount of S100^+^ processes and the numerous astrocyte processes filled by intermediate filaments revealed by electron microscopy. On the other hand, in the extracellular environment S100 protein may be involved in the positive chemotactic attraction of the high quantity of microglia/macrophages described in the tench ONH [17] and prevent neuronal death and promote axon growth.

Interestingly, we describe for the first time an S100^+^/GFAP^+^/GS^+^ astrocyte ring located in the posterior part of the ONH and strongly activated after injury. These astrocytes may be involved in the reorganization of the regenerating axons, ensuring their entrance to the optic nerve and starting to pack them into bundles [17], [34], [44]. This ring has a similar location to the lamina cribosa in mammals and birds [45].

### Molecular characterization of goldfish *pax2a*

Here we identify two partial transcripts of goldfish *pax2a*, which are highly homologous to the ones identified in zebrafish. We analyze its predicted protein sequences and demonstrate that both goldfish isoforms Pax2a are orthologous to other identified vertebrate Pax2. *Pax2* splicing has been identified in different vertebrates such as zebrafish [29], African clawed frog (*Xenopus laevis*) [46], chicken (*Gallus gallus*) [20], mouse (*Mus musculus*) [47], [48], [49] and human (*Homo sapiens*) [50], [51], [52], however, little is known about the functions of the transcript variants.

Moreover, we demonstrate by RT-PCR that the *pax2a_tv2* isoform is also expressed in goldfish retina, but its expression level is less intense than in the ONH. Although we have not detected the Pax2 protein in the retina, it has been recently reported that Müller cells in chicken and zebrafish central retina express it [18], [20]. We hypothesized that the retina may contain Pax2 but the sensitivity of antibody used in the immunohistochemical assays could be too low to detect very low amounts of the Pax2a protein.

### Expression analysis of goldfish Pax2a in the ONH

It has been proposed that *pax* genes may play an important role during regeneration processes [53]. While *pax6* expression in the retina has been analyzed by RT-qPCR after PGZ cryolesion in goldfish and tench [54] and by western blot after optic nerve crush in zebrafish and a lizard (Ornate Crevice-dragon, *Ctenophorus ornatos*) [55]; there are no previous studies that analyze *pax2* expression after injury and regeneration. Here we show that the *pax2a* gene expression in the ONH is highly affected during regeneration after PGZ lesion, suggesting that it plays an important role in this process. During the process of development of the visual system there is a mechanism of reciprocal inhibition between Pax6 and Pax2 that determines their specific location in the retina and optic nerve [56], [57]. When we compare the *pax6* gene expression level in the goldfish cryolesioned retina [54] with the *pax2a* gene expression level in the ONH, we find that the expression level of *pax6* increases when *pax2a* expression decreases at later regeneration stages. It seems likely that the inhibitory mechanism can be conserved in adult fish and may be actively working during regeneration. Upstream and downstream elements involved in the *pax2a* pathway during visual system development [24], such as retinoic acid [58] or Netrin-1 [59], are affected during regeneration. This suggests that the whole signaling network could be maintained in models of continuous growth and regeneration.

Rodger et al. [55] proposed that Pax6 inhibition in zebrafish retina after optic nerve crush is related with the spontaneous regeneration of RGC axons. The maximum *pax2a* expression level we detected coincides with the arrival of the first regenerating axons to the ONH. Previous studies during visual system development in mice and zebrafish lacking the *pax2* expression [25], [27] and *pax2* over expression in chicken [41], demonstrate that Pax2 is responsible for an appropriate RGC axon guidance throughout the optic nerve. Thus, it seems likely that Pax2 is participating in the axon guidance during regeneration. On the other hand, the increase of *pax6* expression [54] and the decrease of *pax2a* expression in the latest regenerative stages appear to be involved with the restoration of topographic connections [55] when the regenerating axons arrive at the optic tectum [60].

### Dividing cells in the ONH in cryolesioned animals

We find that Pax2^+^/PCNA^+^ cells are particularly located in the posterior part of the ONH close to the retina, as they are in control animals [19], and are activated after injury forming a ring. This new source of astroblasts in the visual system has only been described during mammal development [35], [61]. Thus, we demonstrate for the first time this new astroblast source, present in adult vertebrate control animals [19], which becomes more active in injured animals, and participates in both continuous growth and regeneration processes.

With immunogold labeling we show that Pax2 is not only present in mature astrocytes, but also in the nucleus of immature astrocytes. In both Pax2^+^ and Pax2^−^dividing and immature cells we find doughnut-shaped mitochondria. This particular kind of mitochondria has been described in the cytoplasm of astrocytes in rat hippocampus (http://synapses.clm.utexas.edu/atlas/1_1_4_9.stm). However, nothing is known about their significance. We hypothesize that they could be related with plasticity mechanisms.

Due to the high proliferation rate in the ONH after injury, especially the first post-lesion days, Pax2 may have similar functions as during development, such as promoting Pax2^+^ cell survival [62], assuring their astrocyte identity [18], [20], [25], [27], [63] and preventing their differentiation into pigmentary epithelium [25], [27].

## Materials and Methods

### Animals

For immunohistochemistry (IHC) and PCR analyses we employed adult goldfish (*C. auratus*), 8–12 cm in body length obtained from commercial suppliers. Also, for PCR experiments we used ∼200 zebrafish embryos (*D. rerio*, AB wild type strain) of 5 days post-fertilization (dpf). Goldfish were kept in aquaria at 18±1°C and zebrafish at 28.5±1°C, all of them in a 12 h light/dark cycle and, prior to analysis, they were deeply anesthetized with 0.03% tricaine methanesulfonate in water (MS-222; Sigma).

All procedures used in this work were in accordance with the guidelines of the European communities Council Directive (86/609/EEC and 2003/65/EC) and current Spanish legislation for the use and care of animals (RD 1201/2005). Full details of the study were approved by the Bioethics Committee of Salamanca University (CBE/30/07/08).

### PGZ-cryolesion

The cryo-elimination of the goldfish retina PGZ was carried out in 68 goldfish as described by Lillo et al. [64] and were later sacrificed at survival times of 2, 7, 15, 21, 30, 60, 90 and 120 days post-injury (d).

### Immunohistochemistry

For the IHC experiments in sections, 32 animals were perfused transcardially with a fixative solution containing 4% paraformaldehyde and 0.2% picric acid in phosphate buffer 0.1 M, pH 7.4 (PB:Na_2_HPO_4_ 0.1 M, NaH_2_PO_4_ 0.1 M diluted in distilled water). The eyes were dissected out and post-fixed for 2 h at room temperature (RT) in the same solution. The eyes were cryoprotected in 50% sucrose, embedded in OCT and 14-µm sections were cut on a cryostat. For the IHC over *in toto* retinas, they were first dissected out from 20 animals and fixed for 12 h at 4°C in the same fixative solution.

After washes with phosphate-buffered saline 0.1 M pH 7.4 (PBS: 8 g NaCl, 0.2 g KCl, 1.44 g Na_2_HPO_4_, 0.21 g KH_2_PO_4_ diluted in distilled water) with 0.02% Triton 100 (PBS-Tx), sections and retinas were post-fixed with 100% methanol for 5 min and auto-fluorescence was quenched with 2.5 g/L NaBH_4_ in PBS. They were blocked with 2% normal goat serum (2 h): the sections were later incubated overnight at RT with the primary antibodies (Table 1) diluted in PBS-Tx-serum with 1% dimethylsulfoxide (DMSO, Sigma) and the *in toto* retinas were incubated for 4 days at 4°C. After washes, sections and retinas were incubated with 1∶250 fluorescent secondary antibodies Cy2 and Cy3 (Jackson). PCNA labeling was enhanced using a biotinylated antibody (Vector) (1∶250) and then streptavidin Cy2 (Jackson) (1∶200). Nuclei were labeled with DAPI (Sigma) diluted 1∶10,000. Negative controls without first or secondary antibodies were performed. Sections and *in toto* retinas were mounted with an anti-fading mixture [19].

**Table 1:** Antibodies used for immunohistochemistry assays.

Antibody	Host	Description	Supplier	Catalogue number	Dilution
Cytokeratin	Mouse monoclonal	Mixture of monoclonal antibodies from the following clones: C-11, PCK-26, CY-90, KS- 1A3, M20, and A53-B/A2.	Sigma	C2562	1∶100
GFAP	Mouse monoclonal	Derived from the hybridoma G-A-5 produced by the fusion of mouse myeloma cells and splenocytes from BALB/c mice immunized with purified GFAP from pig spinal cord.	Sigma	G6171	1∶400
Pax2	Rabbit polyclonal	Pax2 sequence corresponding to amino acids 188–385 (Pax2A and Pax2B)	Covance	PRB-276P	1∶900 (fluorescence)1∶200 (electron microscopy)
PCNA	Mouse monoclonal	Rat PCNA made in the protein A expression vector pR1T2T	Santa Cruz Biotech.	Sc-56	1∶500
S100	Rabbit polyclonal	S100 isolated from bovine brain.	Dako	Z0311	1∶1500
Zn8	Mouse monoclonal	Neuronal cell surface marker (SC-1, DM-GRASP, BEN)<INPUT VALUE = \neuronal cell surface marker (SC-1, DM-GRASP, BEN) NAME = custcol_antigen TYPE = hidden>§MACROBUTTON HTMLDirect	Hybridoma Bank		1∶300
ZO1	Mouse monoclonal	Human recombinant ZO1 fusion protein encompassing amino acids 334–634.	Invitrogen	33–9100	1∶100

For the statistical analysis of the number of cells in the ONH, we counted cells from 2–6 tissue sections randomly selected from four control and cryolesioned animals of each survival time analyzed (2–120 d). We counted all labeled cells from the area located between the two edges of the neural retina near the ONH excluding the central artery (CA) [19]. For the statistical analysis of the number of cells in the retina, we divided the *in toto* retina in three parts: PGZ, intermediate zone and central retina close to the ONH and we counted cells of each part considered from the four quadrants of the *in toto* retina, measuring 544,000 µm^2^ each. We used 6 control and 6 cryolesioned animals of 7, 21 and 30 d post-lesion. All cell counts were performed with ImageJ software (http://rsb.info.nih.gov/ij/index.html). The statistical analysis was performed using ANOVA followed by the *post-hoc* Bonferroni test to compare different regions in the same experimental group and the *post-hoc* Dunnett's t-test to compare animals with different survival times with control ones.

### Immunogold labeling

Six animals were transcardially perfused with a fixative solution containing 4% paraformaldehyde, 0.2% picric acid and 0.25% glutaraldehyde in PB. The ONH were dissected out and post-fixed with 4% paraformaldehyde and 0.25% glutaraldehyde in PB for 2 h at RT. After several washes in PB, the ONH were cryoprotected in 25% sucrose and 10% glycerol in PB, frozen and thawed three times and washed with PB. The immunostaining was performed following the protocol described by Parrilla et al. [19]. The concentration of the primary anti-Pax2 antibody is indicated in Table 1 and the ultra-small gold-conjugated goat anti-rabbit secondary antibody (Aurion, NL) was diluted 1∶100. Afterwards, the ONH were fixed with 0.5% osmium tetroxide (EMS) for 20 min at RT, dehydrated at 4°C, and flat-embedded in Epon 12 resin (EMS). Ultrathin sections were stained with 2% aqueous uranyl acetate and lead citrate.

### Photomicrographs

Some of the light microscopy images were obtained with an Olympus Apogee digital camera coupled to an Olympus AX-70 photomicroscope and the rest of the fluorescent images were obtained with a laser scanning spectral confocal microscope (Leica TCS SP2). In order to evaluate co-localizations, the same exposure and confocal settings were maintained for each fluorescent channel in the optical and confocal microscope respectively. The ultrathin sections were observed in a Zeiss EM900 electron microscope and the pictures were taken with the coupled digital camera using ImageSP software. Original pictures were further processed with Adobe Photoshop CS4 software.

### Isolation of RNA, RT-PCR and expression analysis

Optic nerves, ONH and retina from 24 adult goldfish and 200 whole zebrafish embryos of 5 dpf were mechanically homogenized separately. Each sample of total RNA was extracted in accordance with the RNeasy® Mini Kit protocol (Qiagen) and purified from genomic DNA with the TURBO DNA-free™ Kit (Applied Biosystems). The quantification of RNA and posterior cDNA was carried out using a nanoPhotometer (Implen GmbH).

Total RNA, primed with random primers, was reverse-transcribed into cDNA using the first-strand cDNA synthesis kit (ImProm-II Reverse-Transcriptase System; Promega) in accordance with the manufacturer's instructions. An RNA-free (negative) control sample was used in these experiments.

In order to identify the sequence of goldfish *pax2* mRNA by PCR assays, we used 25 µl PCR mixture containing 50 ng of cDNA template, 20 pmol of each pair of primers (Table 2), 0.2 mM dNTPs, 1.5 mM MgCl_2_ and 5 units of GoTaq Flexi DNA polymerase (Promega). We used the zebrafish *pax2a* interexonic primers designed by Pfeffer et al. [22]. Also, zebrafish *β-actin* primers, taken from Cid [54] (Table 2) were used as an internal and loading control. PCR amplifications were as follows: 1 cycle at 95°C for 5 min as an initial denaturation step, denaturation at 95°C for 30 s, annealing at 58–63°C for 1 min and extension at 72°C for 1 min (35 cycles) followed by further incubation for 7 min at 72°C. The PCR products were loaded on 2% agarose gels in 40 mM Tris-acetate, 1 m Methylenediamine tetraacetic acid pH 8.0 and visualised by ethidium bromide staining. A DNA free (negative) control sample was used which did not produce any amplified bands.

**Table 2:** Primers used in PCR assays.

Target	GeneBank Number	Primer sequence 5′-3′	Gene position
Pax2a	NM_131184 dr	F: GCAGCCTTTCCACCCATCCTC	933–953
		R: GCTCTGGCTTGATGTGTTCTG	1291–1271
Pax2a_tv2		F: CCCGTGGGGTCCTACTCTAT	100–119
		R: AGCCATCTGAACCATCATCA	171–190
β-actin	NM_131031 dr	F: ACGACCCAGACATCAGGGAG	161–180
		R: CCTCTCTTGCTCTGAGCCTCA	241–221
EF1α	AB056104.1 ca	F: ATGGTGACAACATGCTGGAG	625–644
		R: TCCAGGGCATCAAGAAGAGT	714–733
18 s rRNA	EF189737 ca	F: ATGGCCGTTCTTAGTTGGTG	991
		R: AACGCCACTTGTCCCTCTAA	1118

### Sequencing and phylogenetic analysis

Some PCR products were extracted and purified from the gel using a QIAquick Gel Extraction Kit (Qiagen) following the manufacturer's indications. The PCR product (50–150 ng) and 3 pmol of the sequenced primer (Table 2) were used for sequencing reactions which were performed in a 3100 genetic Analyzer (Applied Biosystems). DNA sequencing was performed on both strands from 2 different animals. DNA sequences were analyzed with Chromas 2.3® software (School of Health Science, Griffith University, Australia) and compared with other nucleotide and/or protein sequence databases using the FASTA and BLAST programs from the European Molecular Biology Laboratory (EMBL; http://www.ebi.ac.uk/embl) and from the National Centerfor Biotechnology Information (NCBI; http://www.ncbi.nlm.nih.gov). The retrieved DNA and protein sequences were aligned with the ClustalW program, setting all the parameters as default [65]. The obtained multiple sequence alignments were then used to construct a neighbour-joining (NJ) tree with MEGA4.0.2 (Molecular Evolutionary Genetics Analysis) software [66] to analyze *p*-distance (calculating the proportion of amino acid differences) with the following parameters: complete deletion and considering a bootstrap value of 1000 replicates. The other settings were given as default by the program.

### Quantitative reverse transcription real time PCR

In order to quantify the gene expression level of goldfish *pax2a_tv2* in the ONH during regeneration by RT-qPCR assays, we used the RNA samples retrotranscribed into cDNA samples obtained from the ONH of 3 control goldfish pooled and 3 cryolesioned animals pooled from 2 to 90 d post-lesion. Goldfish *EF1α* and *18 s rRNA* endogenous genes were used as housekeeping genes [67]. Primers for qPCR (Table 2) were designed based on the *pax2a_tv2* sequence obtained by us and on the available sequence for goldfish *EF1α* gene in the Genebank database (Table 2), with the help of OligoPerfect™ Designer software (Invitrogen); *18 s rRNA* primer sequences were taken from Cid [54]. qPCRs were performed using the SYBR-Green method with a 2× Master Mix (Applied Biosystems). Each reaction contained 10 µL of Master Mix, 0.4 µL of each pair of primers (Table 2), 1–3 µL of each cDNA sample in a different serial cDNA quantity for each gene, and MilliQ water up to 20 µl. The amplification reaction took place in an ABI Prism 7300 detection system (Applied Biosystems), with the following conditions: 10 min at 95°C followed by 40 cycles of 15 s at 95°C and 1 min at 60–63°C depending on each pair of primers. Three PCR reactions were performed for each sample per plate, and each experiment was repeated twice.

The comparative threshold cycle (Ct) method was used for presenting quantitative data [68]. Following the removal of outliers, raw fluorescence data were used to determine the PCR amplification efficiency (E) according to the formula E = [10^(−1/slope)^−1]*100. All amplifications had an E value of 100±10% the E value close to 100% being an indicator of efficient amplification. The relative gene expression value (“fold change”) for each transcript was calculated according to the equation E^−(ΔCt “condition 1”−ΔCt “condition 2”)^, where “condition 1” corresponds to experimental samples (1–90 d), “condition 2” to samples of control animals and ΔCt of each “condition” is Ct _“experimental gene”_−Ct _“endogenous gene”_ [68], [69]. A standard error for each relative gene expression value was calculated as a measure of data variation. One-way ANOVA and subsequent post hoc Dunnett's t-test were applied to establish the statistical significance of differences.
